# Structure of a bacterial putative acetyltransferase defines the fold of the human *O*-GlcNAcase C-terminal domain

**DOI:** 10.1098/rsob.130021

**Published:** 2013-10

**Authors:** Francesco V. Rao, Alexander W. Schüttelkopf, Helge C. Dorfmueller, Andrew T. Ferenbach, Iva Navratilova, Daan M. F. van Aalten

**Affiliations:** 1 Division of Molecular Microbiology, University of Dundee, Dow Street, Dundee DD1 5EH, UK; 2 Division of Biological Chemistry and Drug Discovery, University of Dundee, Dow Street, Dundee DD1 5EH, UK; 3 MRC Protein Phosphorylation and Ubiquitylation Unit, College of Life Sciences, University of Dundee, Dow Street, Dundee DD1 5EH, UK

**Keywords:** signalling, *O*-GlcNAc, glycobiology, protein structure

## Abstract

The dynamic modification of proteins by *O*-linked *N*-acetylglucosamine (*O*-GlcNAc) is an essential posttranslational modification present in higher eukaryotes. Removal of *O*-GlcNAc is catalysed by *O*-GlcNAcase, a multi-domain enzyme that has been reported to be bifunctional, possessing both glycoside hydrolase and histone acetyltransferase (AT) activity. Insights into the mechanism, protein substrate recognition and inhibition of the hydrolase domain of human OGA (hOGA) have been obtained via the use of the structures of bacterial homologues. However, the molecular basis of AT activity of OGA, which has only been reported *in vitro*, is not presently understood. Here, we describe the crystal structure of a putative acetyltransferase (*Og*pAT) that we identified in the genome of the marine bacterium *Oceanicola granulosus*, showing homology to the hOGA C-terminal AT domain (hOGA-AT)*.* The structure of *Og*pAT in complex with acetyl coenzyme A (AcCoA) reveals that, by homology modelling, hOGA-AT adopts a variant AT fold with a unique loop creating a deep tunnel. The structures, together with mutagenesis and surface plasmon resonance data, reveal that while the bacterial *Og*pAT binds AcCoA, the hOGA-AT does not, as explained by the lack of key residues normally required to bind AcCoA. Thus, the C-terminal domain of hOGA is a catalytically incompetent ‘pseudo’-AT.

## 2. Introduction

Since its discovery three decades ago [1], it has become clear that modification of serine and threonine residues by a single *O*-linked *N*-acetylglucosamine (*O*-GlcNAc) is an essential, abundant and dynamic posttranslational process (reviewed in [2,3]). *O*-GlcNAc-modified proteins have been detected in both the nucleus and cytoplasm of higher eukaryotes [1,4] and it was shown that the levels of *O*-GlcNAc respond to nutrient levels and cellular stress [5]. *O-*GlcNAc shows extensive crosstalk with protein phosphorylation [6]. Modification by *O*-GlcNAc has been detected in hundreds of proteins [7], many of which play key roles in cellular processes. For instance, precise *O*-GlcNAc levels at specific sites on insulin receptor substrate 1 (IRS-1), protein kinase Bβ, glycogen synthase kinase 3β and glycogen synthase are required for proper insulin sensitivity and response [8]. *O*-GlcNAc modification of transcription factors, such as c-Myc [9] and mSin3A [10], directly affects their activity [5]. Recently, it was shown that histones are dynamically modified with *O*-GlcNAc in the nucleosome core, suggesting that *O*-GlcNAc may be part of the histone code [11].

Dynamic protein *O*-GlcNAcylation is achieved by the interplay of two essential enzymes; *O*-GlcNAc transferase (OGT) and *O*-GlcNAcase (OGA) [12–14]. Both enzymes are required for life in the metazoan cell and are highly conserved from *Caenorhabditis elegans* to man [7,15]. The N-terminus of OGT possesses multiple tetratricopeptide repeats motifs that have been shown to be essential for recognition of large protein substrates [13,16,17]. Recent studies have reported the structure of a bacterial OGT homologue [18,19] and the structure of hOGT [20], and two different reaction mechanisms have recently been proposed [21,22].

Human OGA (hOGA) is a 92 kDa multi-domain protein, originally identified as an antigen expressed by meningiomas (MGEA5) [14,23,24]. Bioinformatic [25] and biochemical [26,27] studies have suggested that OGA possesses dual catalytic activity. The N-terminal portion of the enzyme recognizes and hydrolyses *O*-GlcNAc-modified peptides/proteins [26,28] and belongs to the CAZy glycoside hydrolase family 84 (GH84) [29]. The use of structural and biochemical characterization of close bacterial homologues has helped our understanding of how the N-terminal domain of eukaryotic OGA would recognize and process *O*-GlcNAc substrates and has stimulated the development of a number of potent hOGA inhibitors [30–35]. Furthermore, bioinformatic analysis has suggested that the C-terminal domain of hOGA can adopt a GCN5 family acetyltransferase (AT)-like fold [28]. Toleman *et al.* [27] have reported *in vitro* histone AT activity for the C-terminal hOGA domain (hOGA-AT) purified using a mammalian expression system; however, activity for protein purified from bacteria was only observed after incubation with mammalian cell lysate [27,36]. These data prompted the authors to rename hOGA to nuclear cytoplasmic *O*-GlcNAcase and acetyl transferase [27]. The OGA and AT activities have been suggested to act synergistically, opening up the chromatin and directly activating transcription factors [27]. However, a report by Butkinaree *et al*. [37] casts doubt on this, as the authors were unable to reproduce histone AT activity.

There is currently no crystal structure of any eukaryotic OGA. It is thus unknown how hOGA-AT recognizes acetyl-CoA (AcCoA) or possible protein substrates and how this domain is positioned relative to the glycoside hydrolase domain. In addition, the amino acids that are involved in catalysis have not been identified. These interactions are generally poorly conserved within the GCN5 family, which makes predictions from bioinformatics or related structures challenging. Here, using X-ray crystallography and structure-guided mutagenesis coupled with surface plasmon resonance (SPR), we provide the first molecular insights into the structure of hOGA-AT by the use of a close bacterial homologue. Our data reveal that while the bacterial homologue binds AcCoA, hOGA-AT does not bind AcCoA owing to amino acid substitutions in the binding site. These results suggest that the C-terminal domain of OGA is a pseudo-AT (pAT) not capable of catalysing acetyl transfer onto a histone substrate.

## 3. Results and discussion

### 3.1. The marine bacterium *Oceanicola granulosus* possesses an operon-containing OGA-AT-like proteins

**Figure 1.**
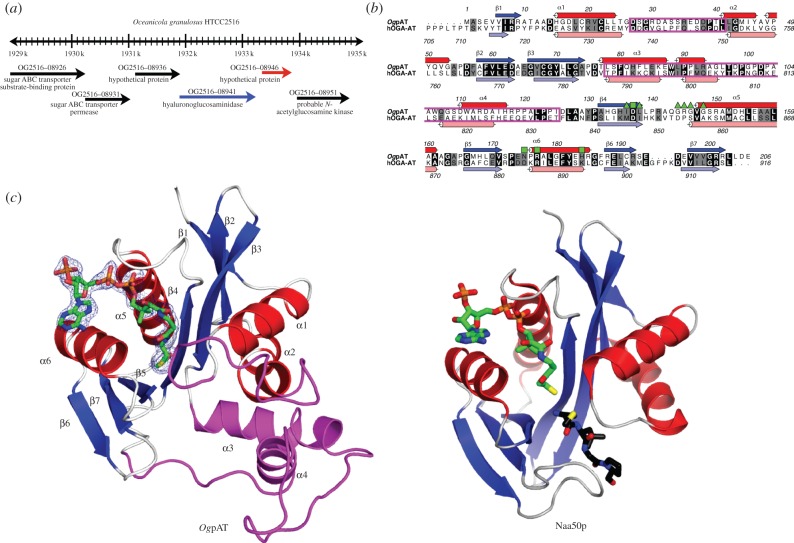
(*a*) Location of *Og*OGA (blue) and *Og*pAT (red) in the *Oceanicola granulosus* genome. (*b*) Sequence alignment of *Og*pAT and hOGA-AT. Identical residues are depicted in black. Secondary structure (calculated using DSSP [39]) for *Og*pAT is shown in blue and red for β-strands and α-helices, respectively. Predicted secondary structure elements (calculated using JPred [40] for hOGA-AT are shown in light blue and pink for β-strands and α-helices, respectively. AcCoA-interacting residues of *Og*pAT are indicated by green squares (interaction involves side chains) or green triangles (interaction involves backbone only). The two magenta boxes represent the two insertions when compared to sequences from other GNAT members. Numbering of the sequences are in accordance with their UniProt entries. Sequences were aligned with ClustaW [41] and annotated using the program ALINE [42]. (*c*) Cartoon view (colour based on secondary structure) of *Og*pAT in complex with AcCoA and Naa50p, an N-terminal AT (PDB code 3TFY [43]) in complex with CoA (green carbon atoms). The Naa50P acceptor peptide is shown with black carbon atoms. The two insertion regions in *Og*pAT are depicted in magenta. The unbiased |*F*_o_|–|*F*_c_|, *ϕ*_calc_ electron density map for AcCoA is shown in cyan, contoured at 2.5*σ*.

Despite the efforts by several research groups, full-length and truncated metazoan OGA has so far resisted protein crystallization. Bacterial homologues have previously been used to gain insights into the structure, mechanism and substrate recognition of the metazoan OGA-GH84 catalytic domain [30,32,34]. Of particular interest is a GH84 from the marine bacterium *Oceanicola granulosus*, *Og*OGA, which was recently crystallized [34], as it shows higher sequence identity to hOGA when compared with other bacterial OGA homologues, with sequence conservation extending beyond the catalytic core revealing a conserved peptide-binding groove [34]. Strikingly, close inspection of the *Og*OGA genomic location reveals an open reading frame coding for a predicted AT [25,38] immediately downstream of the *Og*OGA gene (figure 1*a*). Sequence alignment of this predicted AT from *O. granulosus* (*Og*pAT) with the hOGA-AT domain (hOGA-AT) shows good similarity (30% sequence identity; figure 1*b*). Furthermore, secondary structure predictions for hOGA-AT and *Og*pAT using Jpred [40] support that these two domains are structurally similar (figure 1*b*). Overall, the genomic organization of *Og*OGA and *Og*pAT bears remarkable similarity to the domain arrangement in hOGA. The biological functions of *Og*OGA and *Og*pAT in *O. granulosus* are presently unknown and reversible intracellular *O-*GlcNAc modification of proteins has not been detected in bacteria.

### 3.2. *Og*pAT possesses an acetyltransferase-like fold with a conserved AcCoA-binding pocket

To gain insights into the structure and function of hOGA-AT, we selected the bacterial *Og*pAT for structural studies. The gene for *Og*pAT was cloned into pGEX-6P-1 for expression as a GST (glutathione transferase) fusion in *Escherichia coli.* The protein was purified using glutathione affinity and crystallized from ammonium sulfate solutions (see Material and methods). Crystals of *Og*pAT in complex with AcCoA formed in space group P3_2_21 with one molecule in the asymmetric unit and synchrotron diffraction data were collected to 1.8 Å resolution (table 1). The *Og*pAT structure was solved using experimental phases from a tungsten derivative and refined to *R* = 19.9%/*R*_free_ = 23.4% with good stereochemistry (table 1). The crystal packing suggests that *Og*pAT is a monomer in solution.

**Table 1.** *Og*pAT X-ray diffraction data collection and refinement statistics. Values in parentheses pertain to the highest resolution shell of about 0.1 Å. Ramachandran plot values were obtained from PROCHECK [44].

	*Og*pAT-AcCoA complex
space group	*P*3_2_21
cell dimensions	*a* = *b* = 65.06 Å, *c* = 92.01 Å
wavelength	1.85 Å
no. of reflections	21060
resolution (Å)	20.00–1.80
*R*_merge_	0.043 (0.424)
*I*/*σ*(*I*)	16.8 (3.5)
completeness (%)	98.1 (97.9)
redundancy	4.9 (4.8)
*R*_work_/*R*_free_ (%)	19.9/23.4
protein residues	202
ligand molecules	001
solvent molecules	139
〈*B*〉(Å^2^):	
protein	29.9
ligand	39.1
solvent	40.2
r.m.s. deviations:	
bond lengths (Å)	0.014
bond angles (°)	1.54
Ramachandran fit:	
most favoured (%)	91.4
allowed (%)	08.6
disallowed (%)	00.0

The structure of *Og*pAT consists of mixed α/β-fold, composed of a central seven-stranded twisted β-sheet (topology 1234576) sandwiched between six α-helices (two packed on one side of the β-core and four on the other; figure 1*c*). The β-sheet comprises mostly antiparallel strands except for strands four and five, which are parallel, resulting in a V-shaped wedge appearance, a feature shared with other acetyl transferases (figure 1*c*) [43,45]. The structure reveals a typical conserved AcCoA-binding region composed of one α-helix (α5) and five β-strands (β1–5) as shown in figure 1*c*. Despite low sequence homology, the overall fold identifies *Og*pAT as a member of the GCN5-related *N*-AT (GNAT) superfamily [46]. Members of the GNAT family share a conserved mechanism of catalysis involving an active site carboxylate (reviewed in [47]). Structural homology searches using SSM [48] reveals several members of the GNAT family that share structural features with *Og*pAT. Two of the most similar structures are the amino terminal AT Naa50p and RimI [43,45], with a r.m.s. deviation of Cα atoms of 2.2 and 2.0 Å (for 137 and 132 residues), respectively. The structures of *Og*pAT and Naa50p are compared in figure 1*c*, showing that much of the α/β core is conserved. However, the greatest region of divergence between the two proteins is that *Og*pAT contains an elongation of the loop between α1 and α2 (residues 27–41) and two additional helices (α3 and α4) are inserted between strands β3 and β4 (residues 78–125; figure 1*c*). These insertions create a narrow tunnel-like structure above the presumed catalytic site of *Og*pAT, which is likely to determine the acceptor specificity of the protein. These two insertions are not present in any of the known homologous GNAT structures, and thus appear unique to the *Og*pAT structure. Sequence comparison with other eukaryotic OGA-AT domain shows that this insertion is also present (figure 1*b*).

**Figure 2.**
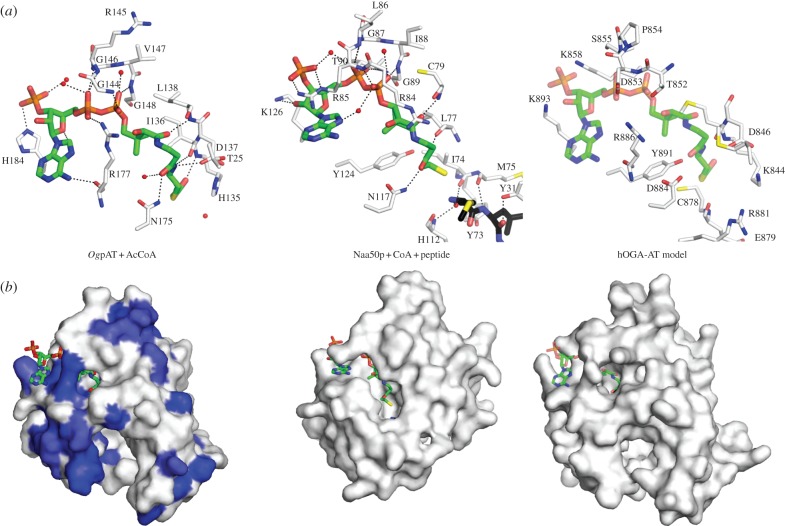
The *Og*pAT-AcCoA complex is compared with the Naa50p-CoA-peptide complex [43] and a model of hOGA-AT in complex with a superimposed AcCoA, using stick model views of the active site (*a*) and molecular surfaces (*b*). CoA/AcCoA are shown as sticks with green carbon atoms. Red spheres represent water molecules. Hydrogen bonds are shown as black dotted lines. The *Og*pAT surface is coloured by similarity to hOGA-AT (identical residues = dark blue; chemically similar residues = light blue, figure 1*b*).

Inspection of the putative AcCoA-binding site revealed well-defined |*F*_o_|–|*F*_c_|, *ϕ*_calc_ electron density for the ligand, allowing building and refinement of the complete AcCoA molecule (figures 1*c* and 2). The interactions between AcCoA and *Og*pAT are similar to those observed throughout the GNAT superfamily [43]. The adenosine moiety of AcCoA is located on the *Og*pAT surface and stacks against helix α6, while the ribose and 3′-phosphate project into the solvent (figure 2). The 3′-phosphate forms a hydrogen bond interaction with the side chain of His184 located at the end of helix α6. The pyrophosphate and pantetheine moieties form a series of both direct and water-mediated hydrogen bonds to the protein (figure 2). The most conserved interactions between the protein and AcCoA involve the ‘P-loop’ motif [38], which resides at the beginning of helix α5 (figures 1*c* and 2). The ‘P-loop’, which is conserved within the GNAT AT ‘motif A’ [10,38], is crucial for the recognition of the AcCoA pyrophosphate group in all ATs and consists of a conserved sequence [Gln/Arg]-x-x-Gly-x-[Gly/Ala]. In the *Og*pAT-AcCoA complex, the ‘P-loop’ is located at the start of helix α5 (figures 1*b,c* and 2) and consists of residues 143–148 (sequence Gln-Gly-Arg-Gly-Val-Gly). These residues, together with water molecules, form a network of hydrogen bonds with the pyrophosphate group of AcCoA (figure 2).

The thioester group of AcCoA is sandwiched between strands β4 and β5. The side chain of Asn175, which is conserved among the GNAT members, donates a hydrogen bond to the amide oxygen of the CoA pantothenate/cysteamine link, while the NH of the same amide forms a hydrogen bond with the side chain of Asp137 (figure 2). The acetyl group of AcCoA forms a hydrogen bond with the backbone nitrogen of Ile136 (figure 2). This type of interaction, where the carbonyl of the acetyl group is hydrogen bonded to the main-chain amide nitrogen of a residue downstream of the β-bulge on β4, has been observed in complexes of other GNAT proteins such as Naa50p (figure 2). The interactions between β4/β5 and coenzyme A are a distinctive characteristic of GNAT proteins and are an essential structural feature in promoting acetyl transfer. Thus, *Og*pAT is capable of binding AcCoA and sequence conservation in the active site suggests this as an active enzyme with an as yet unidentified acceptor substrate.

### 3.3. hOGA-acetyltransferase is a pseudo-acetyltransferase lacking key residues for catalytic activity and AcCoA binding

Circular dichroism experiments revealed that the secondary structure composition of hOGA-AT, purified by overexpression in *E. coli*, is very similar to that calculated from the *Og*pAT structure (see electronic supplementary material, figure S1). This, combined with the sequence similarity between *Og*pAT and hOGA-AT (30% identity and 45% similarity, figure 1*b*), allowed construction of a homology model of hOGA-AT using SWISS-MODEL [49]. By sequence and structural similarity to *Og*pAT, hOGA-AT is likely to possess the two unique insertions, thus creating a similar deep-binding pocket (figures 1*c* and 2).

Close inspection of the hOGA-AT model reveals a number of key differences from the *Og*pAT-AcCoA complex and other members of the GNAT family (figure 2). Most members of the GNAT family contain a ‘P-loop’ [38], located at the N-terminal end of an α-helix such that the helix dipole supports binding of the negatively charged pyrophosphate of AcCoA. The sequence alignment and model of hOGA-AT reveal that the AT domain does not possess the ‘P-loop’ consensus sequence (figure 1*b*), instead a negatively charged aspartic acid (Asp853) is placed in close proximity to the pyrophosphate-binding site of AcCoA (figure 2). Furthermore, the steric constraints imposed by a proline residue (Pro854) are likely to impede the interaction between the hOGA-AT ‘P-loop’ and the AcCoA pyrophosphate (figure 2). This amino acid substitution is not present in any known active member of the GNAT family [38]. In addition, hOGA-AT Met735 (Thr25 in *Og*pAT) may clash with the pantothenate moiety of AcCoA. Taken together, the structural data and model suggest that hOGA-AT is unlikely to bind AcCoA.

Two distinct reaction mechanisms have been proposed for enzymes of the GNAT family [38,50,51]. The first mechanism involves an active site base that deprotonates the substrate amino group resulting in nucleophilic attack on AcCoA. The acetyl group is transferred from the thioester of AcCoA to the target amino group and an active site acid then donates a proton to the sulfur atom of CoA to bring the reaction to completion [52]. The second catalytic mechanism involves a covalent acetyl-enzyme intermediate via a conserved cysteine [53,54]. Analysis of the *Og*pAT active site and comparison with the ternary complex of Naa50p [43] reveals that His135 is in a position to function in catalysis (figure 2). Consistent with the first reaction mechanism, the imidazole group of this residue may participate in proton extraction from the acceptor amine group of the substrate. Subsequently, the uncharged amino group can perform a nucleophilic attack on the carbonyl carbon of the thioester group of AcCoA (figure 2). The residue corresponding to His135 of *Og*pAT, Lys844 in OGA-AT, could possibly act as a base in catalysis, though in the absence of an appropriately activating environment this seems unlikely, this lysine residue appears to be only conserved in OGA-AT of higher eukaryotes. However, inspection of the hOGA-AT model reveals that Glu879 may be a structural equivalent of His112, the general base in the Naa50p structure (figure 2).

It has previously been proposed that Tyr891 acts as a catalytic base and Asp853 or Asp884 as the catalytic acid for the hOGA-AT [27]. From the structure of *Og*pAT, the equivalent residues (Tyr182, Gly144 and Asn175, respectively) are not in a position to participate in catalysis. Tyr182 is more than 9 Å away from any potential acceptor substrate, Gly144 forms part of the conserved ‘P-loop’ and Asn175 forms a hydrogen bond with the pantothenate moiety of AcCoA (figure 2).

**Figure 3.**
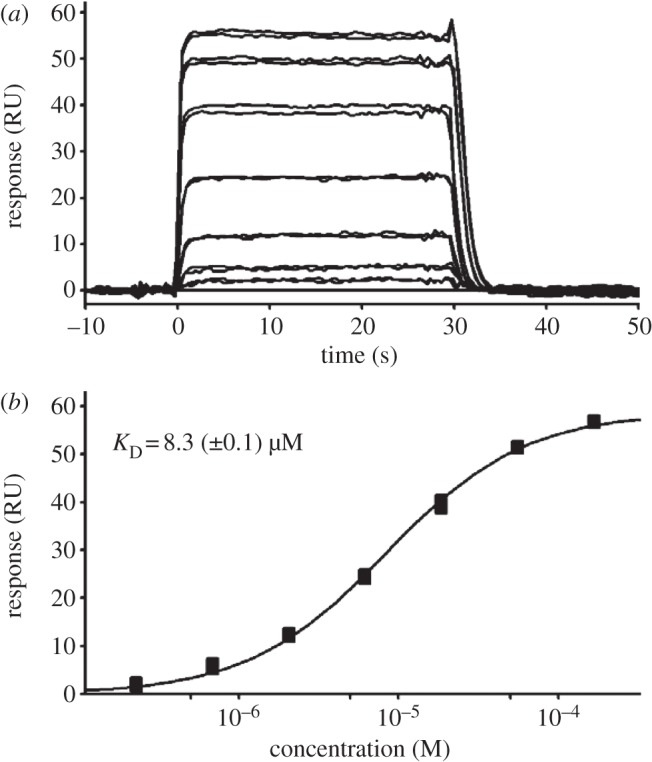
Sensorgram for binding of AcCoA to wild-type *Og*pAT. AcCoA was injected in duplicates at 7 concentrations (0.2, 0.7, 2.1, 6.2, 18.5, 55.5 and 166.7 µM). Equilibrium affinity fit is shown in (*b*) of the figure. RU, relative units.

SPR was used to investigate *Og*pAT and hOGA-AT interactions with AcCoA. As expected from the crystallographic complex, wild-type *Og*pAT binds AcCoA (*K*_d_ = 8.7 μM, figure 3 and table 2). By contrast, wild-type hOGA-AT did not show any detectable binding of AcCoA. Additional SPR experiments with butyryl-CoA, decanoyl-CoA and CoA again revealed no detectable binding to hOGA-AT (table 2) in agreement with the structural analysis of the hOGA-AT model. Furthermore, mass spectrometric experiments showed that hOGA-AT is not purified as a complex with AcCoA from *E. coli*, which would have precluded the detection of AcCoA binding by SPR (see electronic supplementary material, figure S2). To investigate whether the unusual hOGA-AT ‘P-loop’ is compatible with AcCoA binding, key P-loop residues in *Og*pAT (Gly144, Arg145, Gly146) were mutated to the corresponding residues in hOGA-AT (Asp, Pro, Ser) (figures 1*b* and 2). No AcCoA binding could be detected with this *Og*pAT mutant (table 2). The inverse experiment of mutating Asp853, Pro854, Ser855 in hOGA-AT to the *Og*pAT equivalent (Gly, Arg, Gly) resulted in insoluble protein.

**Table 2.** *K*_D_ determination by SPR. n.b denotes no detectable binding.

	*Og*pAT, wt	*Og*pAT, mutant	*h*OGA-AT
AcCoA	8.3 µM	n.b	n.b
butyryl-CoA	—	—	n.b
decanoyl-CoA	—	—	n.b
CoA	—	—	n.b

## 4. Conclusion

The data presented here give the first detailed structural insights into a putative bacterial AT (*Og*pAT) with significant sequence homology to hOGA-AT. The crystal structure of *Og*pAT in complex with AcCoA reveals key amino acids necessary for cofactor binding and gives insights into catalytic residues conserved in other active members of the GNAT family. Based on the *Og*pAT structure, a model of hOGA-AT was constructed and key amino acids were identified. The hOGA-AT model reveals a missing ‘P-loop’ and key amino acids such as Met735 that may impede binding of AcCoA. In addition, the catalytic residues proposed by Kudlow *et al.* [55] do not appear to be in a suitable location to participate in catalysis. The data presented here are not compatible with the original report attributing histone AT activity to this hOGA domain. It is possible that the authors have purified a different protein from BSC-40 cells or a contaminant that had AcCoA transferase activity [27]. This may explain why bacterially expressed hOGA-AT, purified by the same authors, does not show enzymatic activity, which they could only show upon incubation with mammalian cell lysate. The function of the hOGA-AT domain remains unresolved, therefore this domain can be classified as a pseudo-histone AT. The AT domain of OGA is conserved from *Drosophila* to human, suggesting a functional role. Such roles might be simply protein stability of the OGA domain or binding to peptide target sequences to aid localization.

## 5. Material and methods

### 5.1. Cloning, expression and crystallization

A construct encoding hOGA-AT (residues 698–916) was amplified by PCR using gDNA (Sanger Institute, Cambridgeshire). The PCR-product was ligated into the pCR-Blunt II-TOPO (Invitrogen) and subcloned into a modified pET15b plasmid (encoding a PreScission protease (PP) cleavage site instead of the original thrombin site) using the *Nde*I and *Xho*I restriction sites. Site-directed mutagenesis for the hOGA-AT mutants was performed using the QuikChange method (Stratagene) using standard protocols. DNA constructs were verified by DNA sequencing (The Sequencing Service, College of Life Sciences, University of Dundee, Scotland, UK). hOGA-AT-pET15bPP constructs were transformed into *E. coli* ArcticExpress competent cells (Stratagene). Cells were grown overnight at 37°C in Luria-Bertani (LB) medium containing 50 μg ml^−1^ ampicillin. Ten millilitres of the overnight culture was used for inoculation of 1 l LB autoinduction medium and grown at 30°C to reach an OD_600_ of 0.6. The temperature was then reduced to 12°C and cells were grown for 96 h.

The culture harvested by centrifugation for 30 min at 3500 r.p.m. (4°C) and the pellet from 1 l culture was resuspended in lysis buffer A (25 mM Tris–HCl, pH 8.5, 200 mM NaCl, 5 mM DTT) supplemented with protease inhibitors (PMSF (1 mM), leupeptin (0.2 µM) and benzamidine (1 mM)), lysozyme and DNase. The cell pellets were lysed with a constant cell disrupter (three passes at 20 kpsi) and the lysate was cleared by centrifugation (30 min, 20 000 r.p.m., 4°C). The resulting supernatant was passed through a 0.45 µm filter, and loaded onto a 5 ml His-Trap HP column (GE Healthcare) charged with NiSO_4_. The column was washed with 10 column volumes of the same buffer, and subsequently the recombinant protein was eluted applying a linear imidazole gradient (0–500 mM imidazole) over 20 column volumes. Late elution fractions were pooled and buffer exchanged by dialysis into buffer A. The N-terminal His_6_ tag was cleaved overnight by PreScission protease followed by a second round of nickel affinity purification. The resulting solution was concentrated to 5 ml and loaded onto a Superdex 75, 26/60 gel filtration column pre-equilibrated in buffer A. Pure fractions were verified by SDS-PAGE, pooled and spin concentrated using a 10 000 MWCO concentrator.

DNA encoding full-length *Og*pAT was PCR-amplified from genomic DNA using KOD DNA polymerase and then ligated into pGEX-6P-1 cut with *Bam*HI and *Eco*RI. For protein expression, the resulting plasmid was transformed into *E. coli* BL21(DE3) pLysS. Cultures were grown to an OD_600_ of approximately 0.7 at 37°C; after induction with 0.25 mM IPTG they were grown at 20°C overnight before harvesting by centrifugation. Cells were resuspended in lysis buffer (25 mM Tris, pH 7.5; 250 mM NaCl) supplemented with lysozyme and DNAse and lysed by sonication; cell debris and unbroken cells were removed by centrifugation, then the cell lysate was incubated with glutathione-sepharose beads for 1.5 h. After extensive washing with lysis buffer, the fusion protein was cleaved on the beads by incubation with GST-tagged PreScission protease at 4°C for 48 h. Cleaved *Og*pAT was eluted with lysis buffer and concentrated before loading onto a Superdex 75, 26/60 column equilibrated in lysis buffer. Appropriate gel filtration fractions were pooled; the protein was then assessed for purity by SDS-PAGE and concentrated to 3.8 mg ml^−1^.

### 5.2. *Og*pAT data collection, structure solution and refinement

Crystals of *Og*pAT in complex with AcCoA (1 mM) were grown by hanging drop vapour diffusion over a reservoir of 0.1 M Tris, pH 8.5; 2.15 M (NH_4_)SO_4_; 0.24% HCl_conc_ at 20°C. For phasing, apo-*Og*pAT crystals were soaked by addition of 20 mM (NH_4_)WS_4_ to an equilibrated microdrop.

Crystals were cryoprotected by soaking in mother liquor supplemented with 20% ethylene glycol before being flash-frozen for data collection at 100 K. All data were processed/scaled with Denzo/Scalepack [56], and further handled with CCP4 software [57]. Data for a WS_4_^2−^ derivative were collected to 1.85 Å and phased using SHELXC/D/E [58] through HKL2MAP [59]. Automated model building with warpNtrace [60] generated a nearly complete model covering 200 of 206 residues. After rebuilding in Coot [61] and refinement with REFMAC5 [62], this model was used for molecular replacement with the AcCoA complex data, which were collected to 1.80 Å. Refinement was initiated immediately revealing well-defined |*F*_o_|–|*F*_c_|, *ϕ*_calc_ electron density for the ligand, which was built in with the help of PRODRG-generated coordinates and topology [63]. Through rounds of model building in Coot and refinement with REFMAC5 the model was improved to a final *R*_free_ value of 23.4% and validated in Coot and PROCHECK [44]. For complete data and model statistics, see table 1. All figures were produced with PyMOL [64].

### 5.3. Surface plasmon resonance

SPR measurements were collected using Biacore 3000 instrument (GE Healthcare). *Og*pAT (WT, M1) was biotinylated by mixing of the protein with amine-binding biotin (Pierce) in 1 : 1 molar ratio [65]**.** Streptavidin was immobilized on a CM5 sensor chip surface by amine coupling. A total of 10 mM Hepes, 150 mM NaCl, pH 7.4 was used as a running buffer for immobilization. The surface was activated by 15 min injection of NHS/EDC followed by injection of SA in 10 mM acetate, pH 4.5 until the required density (approx. 9000 relative units (RU)) was achieved and blocked by 4 min ethanolamine injection at 10 μl min^−1^ at 25°C. Biotinylated *Og*pAT was captured on the streptavidin surface at approximately 2500–4000 RU in running buffer containing 25 mM Tris (pH 7.5), 150 mM NaCl, 1 mM DTT and 0.005% Tween 20. AcCoA was injected in duplicates at threefold concentration series in a range of concentrations 0.2–166.6 μM. Association was measured for 30 s and dissociation for 1 min. All experiments were run at 50 μl min^−1^ at 25°C. All data were referenced for blocked streptavidin surface and blank injections of buffer. Scrubber 2 (BioLogic Software) was used to process and analyse data. Affinities were calculated using 1 : 1 equilibrium-binding fit.

## Supplementary Material

Supplementary material

## Data accessibility

The atomic coordinates and structure factors of AcCoA complex have been deposited with the Protein Data Bank, with accession number 3ZJ0.
