# Supplementary material for: Structure of a bacterial putative acetyltransferase defines the fold of the human O-GlcNAcase C-terminal domain
Source: Open Biol. 2013 Oct;3(10):130021. doi: 10.1098/rsob.130021 (PMC3814719; doi:10.1098/rsob.130021)
Supplement: Supplementary material [file rsob130021supp1.pdf]

SUPPORTING ONLINE MATERIAL FOR

Structure of a bacterial putative acetyltransferase defines the fold of the human O-GlcNAcase C-terminal domain

Francesco V. Rao[1+], Alexander W. Schüttelkopf¶[1+], Helge C. Dorfmueller[1], Andrew T. Ferenbach[1], Iva Navratilova[2] and Daan M. F. van Aalten[1*]

This PDF file includes:

Supplementary Figures 1-2

Supplementary Figure 1

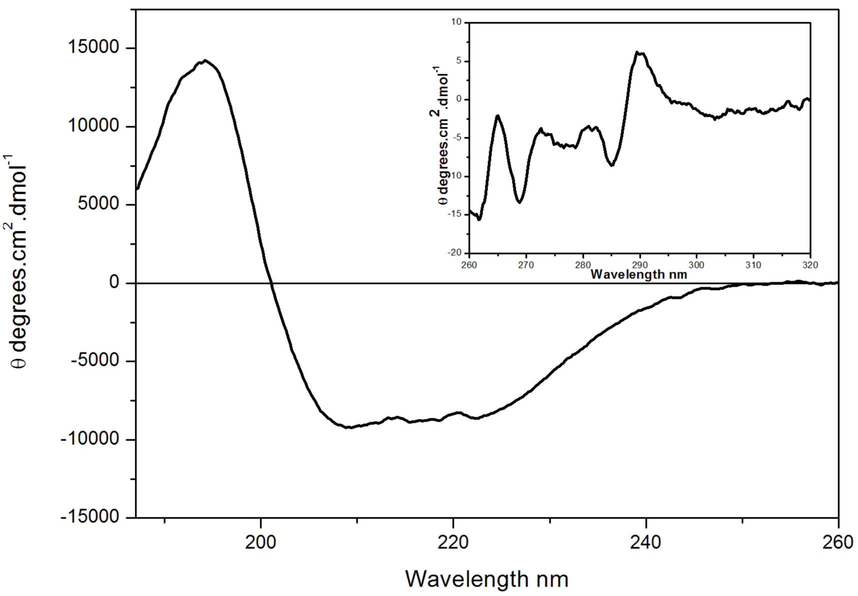

	Helix	Strand
**CD secondary structure**	**30.0%**	**20.7%**
**Calculated based on *Og*pAT structure**	**33.6%**	**20.4%**

Far UV CD (main spectrum) and near UV Circular dichroism (CD) (inset) of human hOGA-AT and calculated secondary structure content.

Spectra were measured in a JASCO J-810 spectropolarimeter using the following parameters: Near UV CD: protein concentration 3.84mg/ml cell; pathlength 0.2 cm; Data pitch 0.2 nm; Band width 1 nm; Response 2 sec; Scanning speed 10 nm/min; no. of scans 3. Far UV CD: protein concentration 0.48mg/ml; cell pathlength 0.02 cm Data pitch 0.2 nm; Band width 1 nm; Response 0.5sec; Scanning speed 50 nm/min; no. of scans 5. Protein was in 10 mM Tris HCl, pH 7.5. Secondary structure estimates were determined using DICHROWEB.

Supplementary Figure 2

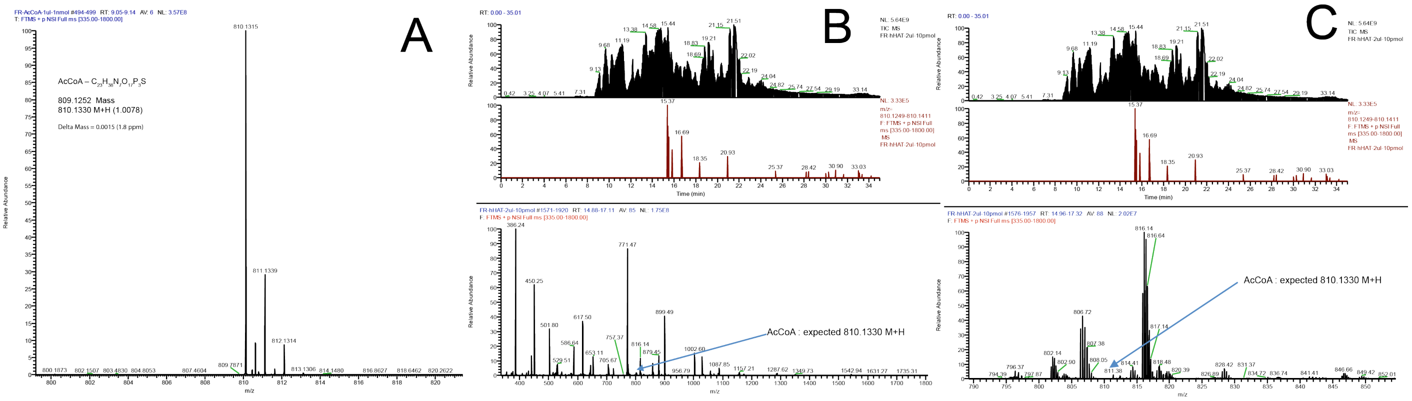

Mass spectrometry analysis of hOGA-AT.

Panel A shows the spectra for the AcCoA standard.

Panel B shows the digest of hOGA-AT indicating eXtracted Ion Chromatogram (XIC) of 810.1330 m/z of AcCoA.

Panel C shows an expanded area of the spectrum in panel B indicating absence of AcCoA m/z.
